# Efficacy and safety of Huangkui capsule for diabetic nephropathy: A protocol for systematic review and meta-analysis

**DOI:** 10.1097/MD.0000000000027569

**Published:** 2021-10-22

**Authors:** Wenrong An, Yanqin Huang, Shouqiang Chen, Tao Teng, Juan Liu, Yunsheng Xu

**Affiliations:** a First College of Clinical Medicine, Shandong University of Traditional Chinese Medicine, Jinan, China; b Department of Endocrine, Affiliated Hospital of Shandong University of Traditional Chinese Medicine, Jinan, China; c Department of Endocrine, Second Affiliated Hospital of Shandong University of Traditional Chinese Medicine, Jinan, China; d Shandong Rehabilitation Research Center, Jinan, China.

**Keywords:** diabetic nephropathy, Huangkui capsule, meta-analysis, protocol, systematic review

## Abstract

**Background:**

Diabetic nephropathy (DN) is 1 of the most serious complications of diabetes mellitus and the leading cause of end-stage renal disease in the world. Huangkui capsule, extracted from *Abelmoschus manihot* (L.) medic (AM), has been widely used to treat DN. However, there is no consensus on the efficacy of Huangkui capsule for DN. This study aims to perform meta-analysis to systematically review the efficacy and safety of Huangkui capsule.

**Methods:**

The following 9 electronic databases will be comprehensively searched: PubMed, web of science, MEDLINE, Embase, Cochrane Library, China National Knowledge Infrastructure, Chinese Scientific Journals Database, Wanfang data, and Chinese BioMedicine Literature Database. The retrieval time is from their inception to May 2021. According to the inclusion and exclusion criteria, 2 reviewers independently completed the study selection, data extraction, risk of bias assessment, and data synthesis. Review Manager Version 5.3 software will be used to conduct meta-analysis.

**Results:**

This study provides a high-quality synthesis to assess the efficacy of Huangkui capsule for treating diabetic nephropathy.

**Conclusion:**

The result of this systematic review will provide objective evidence-based basis to judge the effectiveness and safety of Huangkui capsule on diabetic nephropathy.

## 1 Introduction

Diabetic nephropathy (DN), 1 of the major complications of diabetes mellitus,^[1,2]^ has become a threat to human health and mortality.^[3,4]^ DN shows progressive renal damage over several years and involves many pathological changes: excessive deposition of extracellular matrix, glomerulosclerosis and renal interstitial fibrosis. DN has become 1 of the leading causes of chronic renal failure.^[5]^ Western medicine of DN's clinical treatment is mainly focused on controlling blood glucose level, reducing urine protein, and improving renal microcirculation^[6]^ and often has long-term side-effects. In consideration of its limitations, complementary and alternative medicine may be the prospective options for DN intervention.

Traditional Chinese medicine has obvious curative effect in the prevention and treatment of DN^[7,8]^ and has obtained its popularity in China due to rich theoretical experience, significant advantages, little adverse reactions, and a long history.^[9]^ Among them, Chinese patent medicine Huangkui capsule is a good choice for the treatment of DN. Huangkui capsule, an extract from *Abelmoschus manihot* (L.) medic, gained regulatory approval from China's State Food and Drug Administration (Z19990040) for the treatment of chronic nephritis in 1999.^[10]^ Huangkui capsule has applied to treat DN for about 10 years in clinical practice with no reported adverse reactions.^[11,12]^

No matter from existing clinical studies or animal experiments, Huangkui capsule has been proved to have significant definite effect against DN. Due to small sample sizes, statistical power limitations, insufficient data, and so on, there is no consensus and lack of systematical review on the clinical efficacy of Huangkui capsule in the treatment of DN. Hence, this study aims to perform meta-analysis to systematically review the efficacy and safety of Huangkui capsule, so as to provide reliable and objective evidence-based basis for the clinical application and propose some guides for future research.

## 2 Methods

### 2.1 Study registration

This protocol of systematic review and meta-analysis has been registered on International Platform of Registered Systematic Review and Meta-Analysis Protocols (https://inplasy.com/inplasy-2021-6-0057/.) and registration number is INPLASY202160057.

### 2.2 Inclusion criteria

#### 2.2.1 Types of studies

All randomized controlled trials of Huangkui Capsule alone or combined with other drugs for DN regardless of blinding, allocation concealment will be included. Studies will not be restricted by publication time and area. The language is limited to Chinese and English.

#### 2.2.2 Types of participants

All patients with a clinically diagnosis of DN were included regardless of course of disease, gender, age, race, nationality, education, or economic status.

#### 2.2.3 Types of interventions

The interventions of control group were as follows: conventional drugs (such as hypoglycemic, antihypertensive), placebo or only diet and exercise control treatment. The experimental group was added with Huangkui capsule based on the control group. There is no restriction of drug dosage, time, frequency or duration of treatment in this study.

#### 2.2.4 Types of outcome measures

The primary outcome is efficacy indicators including the total clinical effective rate (calculated by the recovery of biochemical indicators and clinical symptoms), 24-hour urinary total protein, serum creatinine, blood urea nitrogen, urine albumin excretion rate. The secondary outcome is safety indicators, including adverse reaction rate.

### 2.3 Exclusion criteria

#### 2.3.1 Animal studies, review reports, conferences, letters or case reports

#### 2.3.2 Duplicated published data

#### 2.3.3 The full-text literature cannot be obtained or invalid outcomes

### 2.4 Search strategy

#### 2.4.1 Electronic searches

A comprehensive search of electronic databases is carried out from the initiation of the databases to May 2021. Following total 9 English and Chinese databases will be searched: PubMed, web of science, MEDLINE, Embase, Cochrane Library, China National Knowledge Infrastructure, Chinese Scientific Journals Database, Wanfang data, and Chinese BioMedicine Literature Database. Based on the principle of combination of heading terms and free words, 2 reviewers will manually search of the following terms using Boolean logic (AND, OR, or NOT): “Huangkui capsule”, “Huangkui”, “*Abelmoschus manihot*”, “Diabetic nephropathy”, “Diabetic Kidney Disease”, “DN”, “randomized controlled trials”, “randomized controlled trial”. Disagreements will be resolved through a discussion with a third reviewer. Table 1 presents the retrieval strategy in PubMed.

**Table 1:** Search strategy of the PubMed.

Number	Search terms
1	Huangkui capsule [MeSH] [Title/Abstract]
2	Huangkui [Title/Abstract]
3	*Abelmoschus manihot* [Title/Abstract]
4	Diabetic Nephropathy [MeSH] [Title/Abstract]
5	Diabetic Kidney Disease [Title/Abstract]
6	DN[Title/Abstract]
7	RCT[Title/Abstract]
8	Randomized controlled trial [Title/Abstract]
9	1or2or3or4
10	1or2or3or5
11	1or2or3or6
12	1or2or3or4or8
13	1or2or3or4or7

#### 2.4.2 Searching other resources

We will retrieve additional relevant publications by searching reference list.

### 2.5 Study selection and data exaction

#### 2.5.1 Study selection

**Figure 1:**
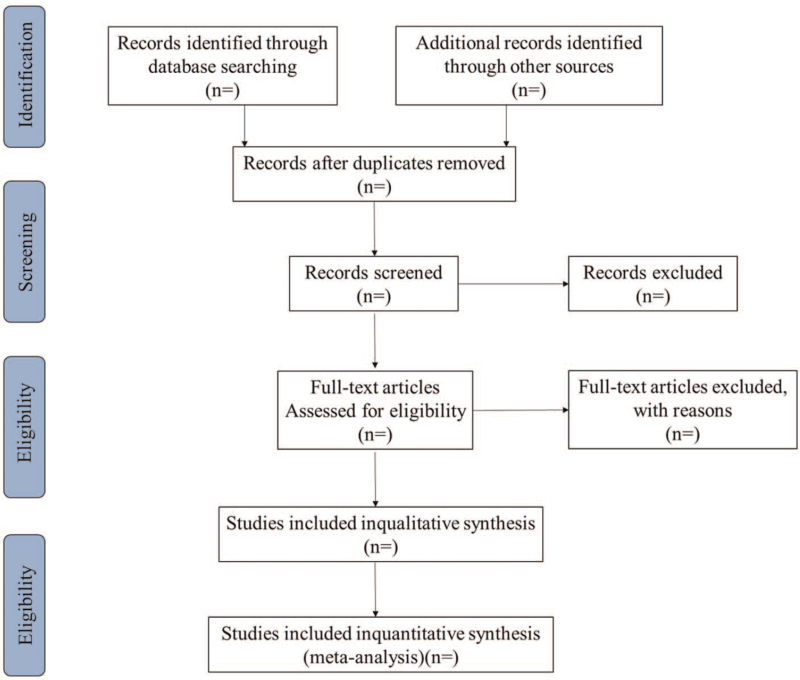
Flow chart of the screening process.

All literatures will be imported into the citation management software Endnote X9 (Thomson Corporation, Stanford, CA) to remove duplicated or ineligible studies. Firstly, 2 reviewers independently conduct a preliminary literature screening by reading the title and abstract according to the inclusion criteria. Then, the full text of all including articles will be thoughtfully evaluated to further determine whether to include or not. Finally, all the final included literature were cross-checked repeatedly by reviewers. Any discrepancies in the selection process will be discussed and resolved with the third reviewers. Flow chart of the screening process is tabulated in Figure 1.

#### 2.5.2 Data exaction

The data items for eligible studies were independently extracted by 2 reviewers, collecting the following items: titles, the first and corresponding author, year of publication, country, journal title, clinical diagnostic information, study design, sample size, age of participants, intervention method, outcome, treatment time, duration, follow-up, and adverse events, etc. The authors of included studies will be contacted for detailed and integrated information when necessary. Inconsistency will be settled after 3 reviewers’ discussion.

### 2.6 Risk of bias assessment

Cochrane Collaboration's tool 5.1.0. will be used to examine the risk of bias. Two reviewers separately conduct the evaluation from the following 7 domains:

- Randomized sequence generation;
- Allocation sequence concealment;
- Blinding of participants and personnel;
- Blinding of outcome assessment;
- Incomplete outcome data;
- Selective outcome reporting;
- Other biases.

A bias value of “L (low risk)”, “U (unclear risk)” or “H (high risk)” will be adopted to rank the risk of bias. In the absence of consensus, the final decision is made by the third reviewer.

### 2.7 Data synthesis and analysis

#### 2.7.1 Data synthesis

The meta-analysis will be performed with Review Manager Version 5.4 software (The Cochrane Collaboration, Copenhagen, Denmark). The mean difference (MD) or standardized mean differences will be to adopted measure the effect for continuous variables. Risk ratios (RR) or odds ratio (OR) will be assumed to calculate the curative effect for dichotomous variables, with both having 95% confidence intervals (95% CI). *I*^*2*^ test is be used to determine the heterogeneity. The study was considered to be homogeneous when *I*^*2*^ ≤ 50%, *P* ≥ .1, a fixed effect model is selected for meta-analysis. Otherwise, If *I*^*2*^ > 50%, *P* < .1, there was significant statistical heterogeneity in the study and the random-effect model will be used.

#### 2.7.2 Subgroup analysis

Subgroup analysis will be performed if data are available and sufficient, such as different intervention time, different stages of diabetic nephropathy, and different outcomes.

#### 2.7.3 Sensitivity analysis

We will undertake a sensitivity analysis to judge the robustness and stability of the review results. The method is that deleting low quality study one by one based on sample size, the risk of bias, missing data, and methodological quality.^[13]^

#### 2.7.4 Reporting biases assessment

If sufficient studies (>10 studies) are included, funnel plots and Egger linear regression tests will be employed to appraise the possible publication bias.^[14]^

#### 2.7.5 Evidence quality assessment

Grading of Recommendations Assessment, Development, and Evaluation will be used to evaluate the quality of evidence, which are divided into 4 grades: high, medium, low, and very low.^[15]^

#### 2.7.6 Missing data management

In case of missing or a lack of the relevant data, get in touch with the author via email or phone to fulfill information. If accurate data is still unavailable after contacting, these studies will be excluded.

### 2.8 Ethics and dissemination

The present study will use published data and does not require ethics approval.

## 3 Discussion

DN is 1 of the most serious complications in diabetes mellitus patients and the primary cause of end-stage renal disease in the world.^[16]^ Huangkui capsule, as a proprietary Chinese medicine, has been clinically used to treat DN. Huangkui capsule possessed functions of antiplatelet aggregation, reducing blood-lipid, oxy-radical inhibition, prompting the elimination of immune complexes, reducing urinary protein, serum creatinine and urea nitrogen, reducing tubulointerstitial injury, and so on.^[17]^ The main components of HuangKui capsule are the total flavonoids extracted from the flowers of *A manihot* (L.) Medic. It has been reported that Huangkui capsule could improve diabetic nephropathy via activating (PPAR)-α/γ and attenuating endoplasmic reticulum stress in rats ^[18]^; suppress abnormal renal cell proliferation and fibrosis by inhibiting the Klotho-dependent TGF-β1/p38MAPK pathway^[19]^; alleviate renal tubular EMT likely by inhibiting NLRP3 inflammasome activation and TLR4/NF-κB signaling^[20]^; alleviates the early glomerular pathological changes by inhibiting Akt/mTOR/p70S6K signaling activity.^[21]^

Although there has made great progress in the molecular mechanism of Huangkui Capsule for DN, there still exist inconsistences in the effect of Huangkui capsule. Thus, this study presents a systematic review and meta-analysis protocol for comprehensively investigate Huangkui capsule for treating DN. The results of this study will provide latest evidence regarding the effectiveness and safety of Huangkui capsule for clinical practice. However, there are some potential defects in the current paper. Due to the limitation of language ability, we only search English and Chinese literature and may ignore studies or reports in other languages.

## Author contributions

**Conceptualization:** Wenrong An, Juan Liu.

**Funding acquisition:** Yanqin Huang, Yunsheng Xu.

**Methodology:** Shouqiang Chen, Tao Teng.

**Software:** Yanqin Huang, Shouqiang Chen.

**Writing – original draft:** Wenrong An.

**Writing – review & editing:** Juan Liu, Yunsheng Xu.
